# Measuring the health benefits of genome and exome sequencing: a systematic review of economic evaluations

**DOI:** 10.3389/fpubh.2025.1728978

**Published:** 2026-01-09

**Authors:** Marianna Riccio, Annalisa Rosso, Leonardo Maria Siena, Valentina Baccolini, Giuseppe Migliara, Antonio Sciurti, Claudia Isonne, Jessica Iera, Francesco Pierri, Carolina Marzuillo, Corrado De Vito, Giuseppe La Torre, Paolo Villari

**Affiliations:** 1 Department of Public Health and Infectious Diseases, Sapienza University of Rome, Rome, Italy; 2 Department of Translational and Precision Medicine, Sapienza University of Rome, Rome, Italy; 3 Department of Life Sciences, Health, and Health Professions, Link Campus University, Rome, Italy

**Keywords:** genome sequencing (GS), exome sequencing (ES), cost-effectiveness, cost-utility, economic evaluation, systematic review

## Abstract

**Introduction:**

Genome sequencing (GS) and exome sequencing (ES) technologies have gained increasing attention in health economics for evaluating their clinical and public health introduction, but their complexity challenges traditional methods. This systematic review aimed to investigate and discuss full economic evaluations (EEs) of GS and ES in relation to health outcomes, with a focus on methodological issues.

**Methods:**

A systematic search of several databases was carried out (PROSPERO CRD42023430992). Quality was evaluated using the Quality of Health Economic Studies instrument. Key methodological features were investigated, and a narrative synthesis of the findings was performed after grouping studies by testing scope.

**Results:**

Overall, 12 recently published cost-utility analyses (CUAs) were included, assessing the use of GS/ES for guiding targeted therapy in oncology (*N* = 4) or major depressive disorder (*N* = 1), and diagnosing rare genetic diseases (*N* = 7). The findings suggested that GS/ES may be cost-effective for diagnosing rare diseases and may also be cost-effective for treatment guidance under favorable conditions. Methodological rigor tended to be higher in treatment guidance studies, whereas EEs in pediatric diagnostics faced greater challenges. Utility values were largely derived from a common survey using validated multi-attribute utility instruments, and studied on proxy conditions. Variability in perspectives, target populations, and costs limited comparability. To strengthen future EEs, standardized methodologies and long-term, real-world data on clinical and non-clinical benefits are needed.

**Conclusion:**

Traditional CUA approaches are essential to guide the implementation of new technologies, but they should be accommodated or complemented by alternative methods, innovative and comprehensive frameworks that capture the broader value of GS/ES and support their integration into clinical and public health practice.

## 1 Introduction

The growing interest in genomic medicine interventions reflects an increasing need to incorporate economic considerations into real-world implementation strategies and evidence-based policy decision (1). In this evolving landscape, next-generation sequencing (NGS) technologies have garnered substantial attention (2). NGS encompasses various techniques, such as genome sequencing (GS), which decodes the organism’s complete genomic sequence, and exome sequencing (ES), which specifically targets the protein-coding regions (3). These advanced techniques are not only challenging traditional genetic testing paradigms (4) but also raise concerns related to the cost-effectiveness (2). GS and ES have shown significant potential in improving the early diagnosis of rare and inherited diseases, particularly in cases with atypical or non-specific symptoms (5–7). They are also accelerating the discovery of novel genes implicated in various genetic disorders (8, 9). As for oncology, GS and ES facilitate the detection of somatic variants that inform the development of personalized therapies (10, 11). However, despite these advancements, their implementation poses challenges from both clinical and public health perspectives, including complexities in data interpretation, limited workforce capacity, risks of overdiagnosis, and broader ethical and societal concerns (1).

Given this context, the demand for robust economic evidence to support the integration of GS and ES into clinical practice has grown substantially (12–17), but their complex and far-reaching implications drive an ongoing debate on which outcome metrics best capture their true economic value (18–21). Already in 2018, a broad systematic review of economic evaluations (EEs) underscored the limited availability of high-quality evidence in clinical settings and noted that diagnostic yield (DY) - while useful - remains a narrow and insufficient outcome measure to be used for these technologies (12). Moreover, Alam et al. critically examined EEs of GS and ES in pediatric populations, highlighting the need to enhance methodological rigor (13). Recently, Ferket et al. proposed conceptual frameworks to address longstanding methodological challenges in genomic EEs, advocating for the inclusion and modeling of broader health outcomes such as quality-adjusted life years (QALYs) (22). Despite existing limitations in this area (18–21), the incorporation of measures like survival gains and quality of life improvements is still widely recognized as essential for informing resource allocation decisions and ensuring comparability with other health interventions (23). To date, no comprehensive synthesis exists that investigates how health outcomes are defined, measured, and incorporated into full EEs of these technologies. Therefore, the primary aim of this review was to examine the methodological approaches used to quantify and integrate health outcomes in EEs of GS and ES. A secondary objective was to report descriptively findings on cost-effectiveness, however the review was not designed to determine whether GS/ES are cost-effective but rather to assess how health outcomes are measured and incorporated within current evaluation frameworks.

## 2 Methods

This review was conducted in accordance with the Cochrane Handbook for Systematic Reviews (24), the Preferred Reporting Items for Systematic Reviews and Meta-Analyses (PRISMA) Statement (25) and the Center for Reviews and Dissemination guidance on undertaking systematic reviews of economic evaluations (26). The review protocol was registered in PROSPERO under the identifier CRD42023430992. Since primary data collection was not part of this study, ethical review board approval and informed consent were not necessary.

### 2.1 Search strategy and study selection

Two researchers searched PubMed, Scopus and Web of Science databases, as well as specific databases including EconLit, the Center for Reviews and Dissemination (CRD) by the University of York, the International Health Technology Assessment Database, the Cost-Effectiveness Analysis Registry by Center for the Evaluation of Value and Risk in Health (CEVR), the Institute for Clinical and Economic Review Assessments Database, covering publications up to May 31, 2025. The search strategy combined terms related to genomic sequencing (e.g., “genome sequencing,” “exome sequencing,” “genomic sequencing”) with economic evaluation concepts (e.g., “economic evaluation,” “cost-effectiveness,” “cost-utility”). Boolean operators (AND/OR) and truncations were used to capture synonyms and variations in terminology. Reference lists of included articles and relevant reviews were also screened to identify additional studies. The complete search strings for each database are provided in Supplementary Table S1. Duplicate articles were removed, and titles and abstracts were independently screened by two researchers. Studies not meeting the inclusion criteria were excluded. The full-text of each eligible article was examined, resolving disagreements through discussion and noting reasons for exclusion. Studies included in relevant previous reviews (12–17, 20) were assessed against our inclusion criteria.

### 2.2 Inclusion and exclusion criteria

The following eligibility criteria were applied for studies to be included: (a) publication in English or Italian, reflecting the language proficiency of the review team; (b) full EE design, such as CEA and CUA; and (c) modeling of GS and ES technologies across any clinical setting and age group. Only EEs focused on health outcomes were included, regardless of the evaluation perspective (healthcare system or broad societal). We considered health outcomes as defined by Second Panel on Cost-Effectiveness in Health and Medicine (27), namely life years gained (LYGs) and QALYs. Articles were excluded when (a) they reported cost–benefit and cost-consequence analyses, were partial EEs (such as cost-analyses, cost-description studies and cost-outcome descriptions), (b) did not include GS/ES in care pathways or did not apply GS/ES to the human conditions, (c) assessed targeted genes sequencing or NGS-technologies other than GS and ES, and (d) examined the DY, other diagnostic results and any outcomes that did not reflect a direct change in health status. Finally, studies not published in peer-reviewed journals, or not original articles were also excluded. We limited our analysis to CEA and CUA studies, as these approaches enable direct comparison of interventions based on standardized health outcome measures such as QALYs or LYGs. Cost–benefit and cost–consequence analyses were excluded because they use different valuation frameworks that do not allow for such comparability across studies.

### 2.3 Data collection and quality assessment

Each included article was independently evaluated by two reviewers who extracted the main study characteristics using a standardized data abstraction form. Discrepancies were resolved by discussion or by consulting a third researcher. The data extracted included bibliographical details and main characteristics of the studies: author name, publication year, country, type of EE, type of sequencing, disease, target population, scope of sequencing, sources of funding and conclusions on cost effectiveness. Data was also extracted on the main features of the EE methodologies: type of cohort and its size, GS/ES-based strategy, reference or alternative strategy, currency and baseline year of evaluation, time horizon, perspective, discount rate, structure of the model used, type of sensitivity analysis, costs and health outcomes included, source of information on costs and outcomes, basic assumptions and parameters of sensitivity analyses. Quality assessment of all included studies was performed independently by two researchers using the Quality of Health Economic Studies (QHES) checklist (28, 29) Potential differences in the assessors’ results were resolved through discussion and achievement of consensus. The QHES uses a weighted rating system based on 16 questions, with a final QHES score ranging from 0 to 100. Articles were of high quality if the total score was >75. Finally, two reviewers independently performed the risk of bias assessment using the Bias in Economic Evaluation (ECOBIAS) checklist (30). This checklist includes 22 items organized into two sections, one of which specifically addresses key aspects of model-based economic evaluations. Since no validated scoring system exist, each item was scored as follows: 1 point for “yes” (fully met), 0.5 points for “partially yes” (partially met), and 0 points for “no” or “unclear.” Based on the total score, studies were classified into low risk (>70%), moderate risk (50–70%), and high risk (<50%). Any discrepancies in scoring were resolved with discussion.

### 2.4 Data synthesis

A meta-analysis was not possible due to the substantial heterogeneity of the strategies compared, a narrative synthesis was carried out to compare the studies’ features, questions, interventions, methods and outcomes. The data synthesis was performed separately according to the scope of GS/ES-based strategy.

## 3 Results

**Figure 1:**
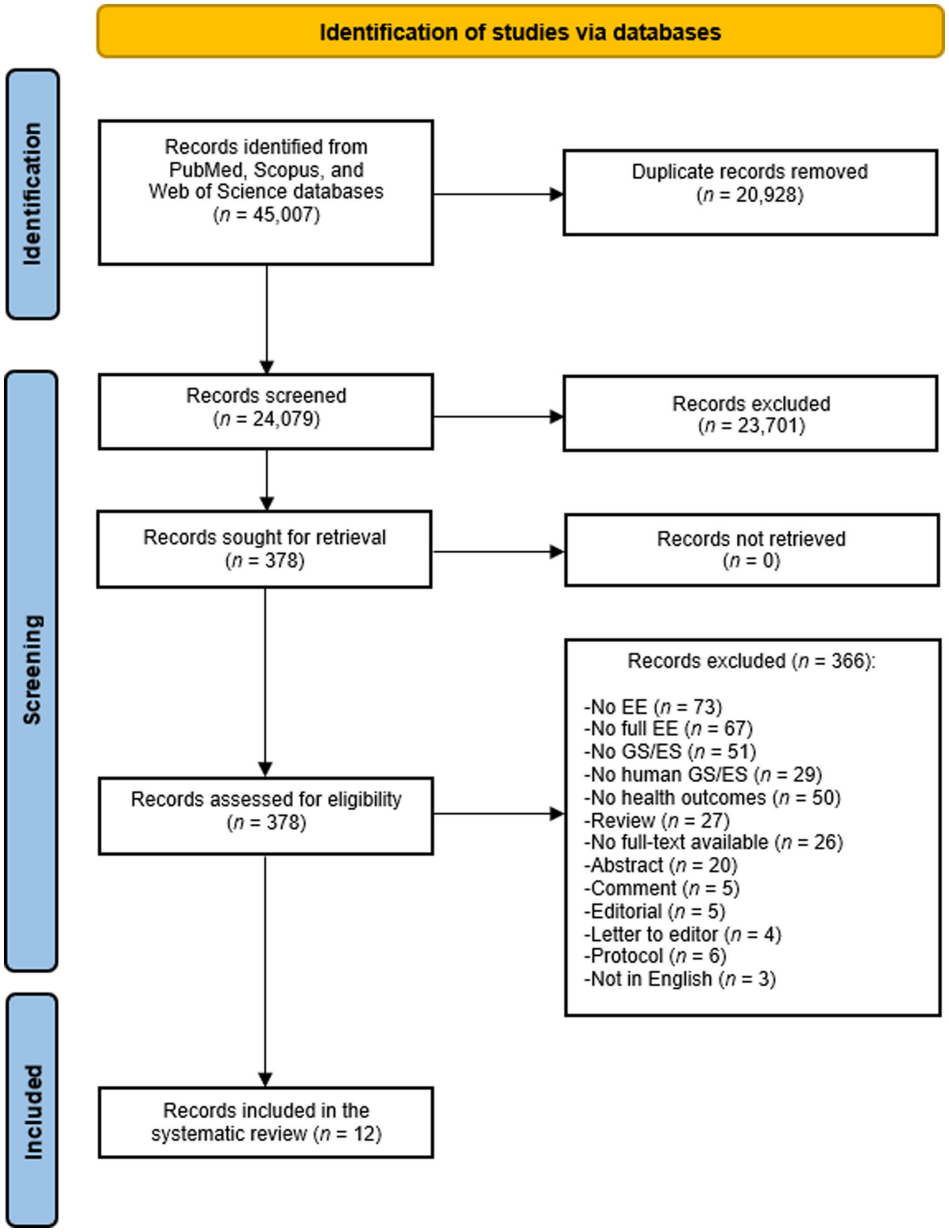
PRISMA flow diagram of the review process.

The database search identified 45,007 articles (Figure 1). After removing duplicates, 24,079 titles and abstracts were screened, yielding 378 full-text articles. Ultimately, 12 studies (31–42) met the inclusion criteria. Included studies were published between 2019 and 2025, four were from the United States of America (38–41), four from the Netherlands (32–35), two from Australia (36, 37), one from the United Kingdom (31) and one from Israel (42). All were CUA, mainly using health economic models, except for two cohort studies (37, 41) reporting real-world data. Four studies (32–35) focused on sequencing cancer treatment decisions and one to guide treatment choices for patients with major depressive disorder (MDD) (31); the remaining seven studies (36–42) examined genetic diagnosis of rare diseases in pediatric populations or during pregnancy.

### 3.1 Main features of the included studies by testing scope

#### 3.1.1 Targeted therapy guidance

Most studies focused on systemic treatment guidance for cancer patients: one study assessed genomic sequencing for metastatic castrate-resistant prostate cancer (35), and three for lung cancer (32–34), focusing on inoperable stage IIIB/IV non-small-cell lung cancer (Table 1). Fabbri et al. (31) modeled the use of ES to assess the risk of pharmacotherapy resistance in patients with major MDD, to guide the choice for psychotherapy alone or in combination with antidepressants. In cancer studies the time horizons ranged from 15 years (35) to lifetime (32–34), with discount rates of 4% (32, 33, 35) and 3% for costs (34), and 1.5% for benefits in all cases. The study on MDD had a 3 years horizon, while discounting was not specified (31). Most studies adopted a societal perspective. Study quality ranged from 92/100 (34) to 100/100 (32, 35) and all studies showed low risk of bias (See Supplementary material).

**Table 1:** Main features of the included economic evaluations, by diagnostic scope of genome/exome sequencing (GS/ES).

First author, year	Country	Type of economic evaluation	Type of sequencing	Disease	Target population	Currency, baseline year of evaluation	Time horizon	Perspective	Discounting	Funding	Quality
Targeted therapy guidance											
Fabbri C, 2020	UK	CUA	ES	Treatment-resistant depression	Patients with major depressive disorder (MDD)	GBP, 2018	3 years	Healthcare	NS	Public, private	93
Simons MJ, 2021	The Netherlands	CUA	GS	Inoperable Stage IIIB, C/IV Non-squamous Non-small-Cell Lung Cancer	Adults aged ≥ 60 yrs. with advanced lung cancer	Euros, 2020	Lifetime	Societal	Costs 4.0% Effects 1.5%	Public, private	100
Simons MJ, 2023	The Netherlands	CUA	GS	Inoperable Stage IIIB, C/IV Non-squamous Non-small-Cell Lung Cancer	Adults aged ≥ 60 yrs. with advanced lung cancer	Euros, 2020	Lifetime	Societal	Costs 4.0% Effects 1.5%	Public, private	94
Mfumbilwa ZA, 2024	The Netherlands	CUA	GS	Advanced non-squamous non-small cell lung cancer ineligible for local treatment.	Adults aged ≥ 40 yrs. with advanced lung cancer	Euros, 2022	Lifetime	Healthcare	Costs 3.0% Effects 1.5%	Public, private	92
Fu J, 2025	The Netherlands	CUA	GS	Metastatic castration-resistant prostate cancer not responding to new androgen pathway targeting agents	Adults aged 70 with metastatic castration-resistant prostate cancer	Euros, 2022	15 years	Societal	Costs 4.0% Effects 1.5%	Not funded	100
Diagnosis of rare genetic diseases											
Schofield D, 2019	Australia	CUA	ES	Rare genetic diseases	Infants aged 0–2 years with suspected conditions	AUD, NS	20 years	Healthcare	Costs: NS Effects 5%	NS	84
Stark Z, 2019	Australia	CUA	ES	Rare genetic diseases	Infants aged 0–2 years with suspected conditions	AUD, NS	NS	Healthcare	Costs: NS Effects 3%	Public	70
Crawford S, 2021	USA	CUA	ES	Mitochondrial diseases	Critically ill newborns	AUD, NS	25 years	Societal	Costs: 3% Effects 3%	NS	91
Avram CM, 2022	USA	CUA	ES	Rare genetic disease causing NHF and fetal effusions	Pregnant women 10–22 GA with NHF and fetal effusions	USD, 2019	Lifetime	Societal	Costs 3.0% Effects 3.0%	Public	93
Lavelle TA, 2022	USA	CUA	GS, ES	Rare genetic diseases	Critically ill infants aged < 1 year, not critically ill children aged < 18 years	USD, 2019	Lifetime	Healthcare	Costs 3.0% Effects 3.0%	Private	94
Sanford Kobayashi E, 2022	USA	CUA	GS	Rare genetic diseases	Critically ill infants/children aged from 4 months to 17 years	USD, NS	NS	NS	NS	Public	60
Friedman MR, 2025	Israel	CUA	ES	Rare genetic diseases	Pregnant women with low-risk pregnancy, at the time of prenatal testing	USD, NS	20 years	NS	Costs 3.0% Effects 3.0%	NS	57

CUA, cost-utility analysis; GA, gestational age; GBP, Great Britain pound; NHF, nonimmune hydrops fetalis; NS, not specified; USD, United States dollars.

#### 3.1.2 Diagnosis of rare genetic diseases

Five studies assessed ES (36–39, 42), one GS (41), and one both (40) (Table 1). Avram et al. (39) studied ES for the diagnosis of genetic disorders linked to fetal effusion and non-immune hydrops fetalis (NIHF) during pregnancy, while Friedman et al. (42) assessed the cost-utility of ES as a tool for prenatal genetic diagnosis in low-risk pregnancy. Crawford et al. (38) focused on mitochondrial diseases (MitD). Target populations included pregnant women (340,43), critically ill newborns (36–38), infants (36, 37, 40, 41), and children in pediatric settings (40, 41). Perspectives varied: healthcare system (36, 37, 40), societal (38, 39), or unspecified. Time horizons ranged from lifetime (39, 40) to 20–25 years (36, 38, 42), and was unspecified in two studies (37, 41). Funding was public private, or unspecified. Study quality scores ranged from 57/100 (42) to 94/100 (41) and risk of bias was low (36, 38–40), moderate (37, 42) and high (41) (See Supplementary material).

### 3.2 Main inputs of the EEs by testing scope

#### 3.2.1 Targeted therapy guidance

The cost of GS was estimated both from commercial sources (2,500€) (32, 33) and literature (3,305–3,218€) (34, 35). ES cost was calculated from literature (388£) (31) (Table 2). The included studies modeled also diagnostic test, follow-up medical visits and treatments costs. Cost estimates were primarily based on published literature and national institutional data. Simons et al. (32, 33) and Fu et al. (35) included indirect costs such as productivity losses (using the friction cost method) and age-dependent medical costs, calculated with the PAID tool v3.0.

**Table 2:** Main inputs of the economic evaluations, by diagnostic scope of genome/exome sequencing (GS/ES).

First author, year	Health costs						Effectiveness measures	
	ES/GS costs	Source	Other health costs	Source	Indirect costs	Source	Health states considered	Source of utility/outcome measures
Targeted therapy guidance								
Fabbri C, 2020	ES £388.5	Literature	Medical visits	Institutional sources	No	NA	Depression, response, remission and death by suicide	Literature (time trade-off and standard gamble studies)
			Psychotherapy					
			Antidepressants					
			Days spent in hospital					
Simons MJ, 2021	GS €2,500	Commercially available test	Diagnostic procedures alternative to GS	Institutional sources, Literature	Productivity, indirect medical	Literature	No progression, progression to first line, progression to second line, and death	Literature (EQ-5D derived utility values; trial data and standard gamble studies on disutilities), assumptions
			Treatment following diagnosis	Institutional sources, literature, assumption				
			Medical visits	Institutional sources				
			Long term care (home care, end of life)	Institutional sources				
Simons MJ, 2023	GS €2,500	Commercially available test	Diagnostic procedures alternative to GS	Institutional sources Literature	Productivity, indirect medical	Literature	No progression, progression to first line, progression to second line, and death	Literature (EQ-5D derived utility values; trial data and standard gamble studies on disutilities), assumptions
			Treatment following diagnosis	Institutional sources Literature				
			Medical visits	Institutional sources				
Mfumbilwa ZA, 2024	GS €3,305	Literature	Diagnostic procedures alternative to GS	Literature	No	NA	No progression, progression to first line, progression to second line, and death	Literature (EQ-5D derived utility values; trial data and standard gamble studies on disutilities), assumptions
			Treatment following diagnosis	Institutional sources Literature				
			Medical visits	Institutional sources Literature				
Fu J 2025	GS €3,218	Literature	Treatment	Literature	Indirect medical	Literature	Complete response/stable disease, progressive disease, death	Literature (EQ-5D derived utility values, trial data for disutilities)
			Long term care					
			Follow up monitoring (Medical visits, lab and diagnostic exams)					
Diagnosis of rare genetic diseases								
Schofield D, 2019	ES AU$3100	Institutional source	Diagnostic procedures alternative to ES	Hospital source Institutional source Literature	No	NA	Levels of disability (NS)	Literature (parental preferences survey: standard gamble and time trade-off; Health Utilities Index Mark 2 for fertility with standard gamble; metanalysis of children utilities)
			Treatment following diagnosis					
			Parental reproductive costs					
Stark Z, 2019	NS	NA	Cascade testing	Hospital source Institutional source Literature	No	NA	Children: health condition at birth Parents: state of fertility	Literature (parental preferences survey: standard gamble and time trade-off; Health Utilities Index Mark 2 for fertility: standard gamble)
			Reproductive services					
			Treatment following diagnosis					
Crawford S, 2021	eES $ 5,247	Literature, expert opinions	Inpatient costs	Institutional sources	Inpatient stay, post discharge	Literature, expert opinions	Within the NICU and post-NICU	Literature (parental preferences survey: standard gamble)
			Long term care (proxy)	Literature				
Avram CM, 2022	ES $2,533	Commercially available test	Diagnostic procedures alternative to ES	Literature	No	NA	Pregnancy: termination, stillbirth, neonatal death. Newborns: mild, moderate or severe outcomes, unaffected neonate	Literature (parental preferences survey: standard gamble), assumptions
			Obstetric and neonatal care (stillbirth, TOP, NND, live preterm)					
			Long term care (proxy)					
Lavelle TA, 2022	rES trio $ 8,112–10,320 rGS trio $10,450–12,000	Literature, Government/institutional sources	Diagnostic procedures alternative to GS/ES	Literature	No	NA	Normal, mild disability, moderate disability, severe disability	Literature (parental preferences survey: standard gamble and time trade-off; SF-6D derived utility values)
Sanford Kobayashi E, 2022	Trio rGS $7,000 Duo rGS $5,700 Proband only rGS $3,900	Research organization	Diagnostic procedures alternative to GS	Hospital sources Literature	No	NA	Death, neurological disability	Literature review and expert opinion (Delphi consensus)
			Treatment					
			Inpatient costs					
Friedman MR, 2025	ES $2,162	NS	Diagnostic procedures (CMA)	Literature	No	NA	Normal results of testing Abnormal results of testing Termination of pregnancy	Clinical data and literature (NS)
			Cost of disability management					

CMA, chromosomal microarray analysis; eES, early exome sequencing; EQ-5D, EuroQol questionnaire; iNMB, incremental net monetary benefit; Lys, life-years; NA, not applicable; NS, not specified; QALYs, quality-adjusted life-years; rGS, rapid genome sequencing; rES, rapid exome sequencing; SF-6D, short-form 6-dimension questionnaire.

Lung cancer studies (32–34) included four health states (no progression, first-line progression, second-line progression, and death), while the prostate cancer study (35) considered complete response/stable disease, progression, and death. The health states considered for MDD were depression, response, remission and death by suicide (31).

Health outcomes were measured in terms of LYGs and QALYs. Transition probabilities, treatment effectiveness, and utility values were primarily derived from published literature relevant to the specific cancer types under study, with disutilities assessed for serious treatment-related adverse events (See Supplementary Table S2). In the lung cancer studies (32–34), as well as in Fu et al. (35), utility values for cancer health states were mainly sourced from patient-reported data in the literature using the EuroQol-5 Dimension (EQ-5D) (43)—a preference-based health-related quality of life (HRQOL) instrument. For adverse events, which were considered only in the oncology studies, most disutility values were drawn from previous cost-effectiveness studies (32–345), standard gamble (31, 33, 34) and time trade off (31, 34, 35) studies and trial data (35). In some cases, these were combined with assumptions specific to the adverse events examined (32–34).

#### 3.2.2 Diagnosis of rare genetic disease

The cost of GS/ES was estimated from various sources, including commercial testing (39), research organizations (41), literature (38, 40), expert opinions (38), and institutional data (36) (Table 2). Costs varied based on sequencing type (rapid, duo, or trio). Schofield et al. also included costs for genetic counseling associated to testing (36). Other health costs varied across studies, including alternative diagnostic procedures (37, 39–42), hospital stays (38, 41), and treatments (36, 38, 41, 42), and some modeled obstetric and neonatal care (40), reproductive services (36, 37), and long-term care for child disabilities (38, 39). Avram et al. (39) used lifetime costs of trisomy 21, 22q11.2 deletion syndrome, and trisomy 13/18 to approximate costs to care for a child with specific postnatal outcomes, while Crawford et al. (37) used cerebral palsy as a proxy for long-term costs of severe MitD. Only Crawford et al. (38) considered indirect costs, calculating unpaid caregiver time for MitD patients, including Neonatal Intensive Care Unit (NICU) stays and home care.

All studies used QALYs as health outcome measures. Avram et al. (39) specifically assessed maternal QALYs, while Stark et al. (37) included QALYs calculated for both patients and first-degree relatives. Generally, pediatric disability levels were used to inform utility calculations in several studies (36, 37, 39–41), and two studies included reproductive outcomes (37, 39) (Table 2). The studies targeting pregnant women (39, 42) also calculated disabilities associated with pregnancy termination following diagnosis. Crawford et al. (38) assessed specifically the utility of NICU hospitalization and a range of post-NICU health states.

Utility values were mainly derived from published literature, either covering a broad range of pediatric health states or focusing on specific conditions used as proxy (See Supplementary Table S2). The parental preferences survey by Carroll et al. (44) using standard gamble and time trade-off methods—using standard gamble and time trade-off methods—was referenced in nearly all included studies (36–38, 40). Additional studies on parental or maternal preferences, incorporating standard gamble methods along with assumptions and findings from previous cost-effectiveness studies, were used in the study by Avram et al. (39). In two studies (36, 37), utilities related to fertility were derived using the validated Health Utilities Index Mark 2 (HUI2) (45), while the Health Utilities Index Mark 3 (HUI3) (46) was applied to post-NICU health states in Crawford et al. (38). Lavelle et al. (40) used utility estimates for comorbidity conditions based on the Short Form-6 Dimension (SF-6D) preference-based measure (47). In one study (41), utility estimates were informed by a literature review of specific pediatric diseases and supplemented with expert consensus via the Delphi method. Lastly, in one study (42) the specific sources related to clinical data and literature were not clearly detailed.

### 3.3 Main inputs of sensitivity analysis by testing scope

#### 3.3.1 Targeted therapy guidance

GS and treatment cost variations were assessed in sensitivity analyses by all studies (Table 3). All studies included the diagnostic accuracy as a parameter in the baseline evaluation, based on the prevalence of known genetic variants, and most also included it as a parameter in sensitivity analyses (31–33). Accuracy data were sourced from literature, expert opinions, and author assumptions. None of the studies included specific considerations on the delivery of GS to targeted populations. Only one study specified that the expected uptake of GS by the target population was 80% (33) in the base model and 100% in all analyses, and included variations in the uptake as a parameter in the sensitivity analyses.

**Table 3:** Main genome/exome sequencing (GS/ES) related inputs of the baseline economic evaluations and sensitivity analyses.

First author, year	Basic assumptions on GS/ES				Parameters included in sensitivity/scenario analyses					
	GS/ES diagnostic yield/accuracy	Source of information	GS/ES uptake	Source of information	GS/ES costs	GS/ES diagnostic yield/accuracy	GS/ES uptake	Treatment costs following GS/ES	GS/ES related utilities	Others
Targeted therapy guidance										
Fabbri C, 2020	NS	NA	No	NA	Yes	Yes	No	Yes	Yes	Cost of visits and hospitalization
Simons MJ, 2021	additional 0.5% of rare molecular targets identified by GS compared to SoCt	Literature Assumptions	NR	NA	Yes	Yes	No	Yes	Yes (effectiveness of treatment)	Cost of SoC
Simons MJ, 2023	additional 0.5% of rare molecular targets identified by GS compared to SoCt	Literature Assumption	80–100%	Assumption	Yes	Yes	Yes	Yes	Yes (progression to second line)	Cot of SoC
Mfumbilwa ZA, 2024	100% specificity and sensitivity for TBM and PDL1	Literature Asssumption	NR	NA	Yes	No	No	Yes	No	Prevalence of TMB-high
Fu J, 2025	No significant incremental differences in accuracy between the two diagnostic strategies	Literature	NR	NA	Yes	Yes (prevalence of genetic variations)	No	Yes	No	Cost of SoC Treatment duration
Diagnosis of rare genetic diseases										
Schofield D, 2019	NA	Real cohort	NA	Real cohort	Yes	No	No	No	No	Later diagnosis/Asymptomatic siblings
Stark Z, 2019	NA	Real cohort	NA	Real cohort	NA	NA	NA	NA	NA	NA
Crawford S, 2021	DY 0.42 (sinlgeton) DY 0.60 (trio)	Literature	NR	NA	Yes	Yes	No	Yes	Yes	Cost of SoC
Avram CM, 2022	DY 0.04 (mild outcomes) DY 0.08 (moderate) DY 0.15 (severe)	Literature	NR	NA	Yes	Yes	No	Yes	Yes	Costs of postnatal/Post-mortem examinations
Lavelle TA, 2022	Infant: DY 0.37 (trio ES) DY 0.49 (trio GS) Children DY 0.28 (trio ES) DY 0.37 (trio GS)	Literature (authors’ calculations)	Infants; 100% rapid trio Children: 100% standard trio	Assumption	Yes	Yes	No	No	No	Cost of SoC After testing costs
Sanford Kobayashi E, 2022	NA	Real cohort	NA	Real cohort	NA	NA	NA	NA	NA	NA
Friedman MR, 2025	DY 0.006	NS	NR	NA	No	Yes	NA	No	Yes (disutilities)	Time horizon Discount rates

DY, diagnostic yield; Yes, item included in the sensitivity analyses; NA, not applicable; NR, not reported; NS, not specified; No, item not included in the sensitivity analyses; SoC, standard of care; TMB, tumor mutational burden.

#### 3.3.2 Diagnosis of rare genetic disease

Some studies performing sensitivity analyses accounted for variations in GS/ES (35, 37–39) and treatment costs (38, 39) (Table 3). Diagnostic yield ranged from a 0.006 probability of positive ES after negative chromosomal microarray (CMA) during pregnancy (42) to 0.49 for trio GS to diagnose rare disorders in infants (40). Lavelle et al. (40) included also expected GS/ES uptake in the baseline model, assuming 100% uptake of rapid sequencing for infants and standard sequencing for children, but omitted it from sensitivity analyses.

### 3.4 Cost-effectiveness of compared diagnostic strategies by testing scope

#### 3.4.1 Targeted therapy guidance

All cancer studies (32–35) calculated the incremental cost-effectiveness ratio (ICER) and incremental net monetary benefit (iNMB) of alternative diagnostic strategies, using a willingness-to-pay (WTP) threshold of €80,000 per QALY (Table 4). The GS-based strategy was generally compared with the standard of care (SoC) to guide the targeted therapy, based on biomarkers testing, including NGS panels and other specific multi-gene panels, Fluorescence *in Situ* Hybridization (FISH), Immunohistochemistry (IHC). Simons et al. (32, 33) used decision tree and transition models to evaluate the cost-effectiveness of GS to guide systemic treatment in lung cancer patients, while Mfumbilwa et al. (34) applied a microsimulation model for immunotherapy selection. Fu et al. (35) used partitioned survival models to guide treatment decision for metastatic castration-resistant prostate cancer (MRPC). Fabbri et al. (31) used a Markov model to calculate the ICER of two different treatment selection strategies for MDD (one based on clinical risk factors only and the other on a combination of clinical and genetic factors, including ES results) *vs* the standard of care of pharmacotherapy to all subjects.

**Table 4:** Features of modeled strategies and conclusions on cost-effectiveness, by diagnostic scope of genome/exome sequencing (GS/ES).

First author, year	Structure of the model	Cohort (size)	GS/ES-based strategies	Reference or alternative strategies	Sensitivity analysis	Conclusions on cost-effectiveness
Targeted therapy guidance						
Fabbri C, 2020	Markov model	Simulated cohort (*n* = 1,000)	Pharmacogenetic + clinical risk-guided (PGx-CL-R) treatment: five clinical factors + rare variants identified by ES and common genetic variants (from genome-wide data) in 83 genes	Clinical risk-guided (CL-R) treatment: five clinical risk factors independently associated with treatment resistant depression (TRD)	Probabilistic	CL-R was more cost-effective than PGx-CL-R PGx-CL-R would become more cost-effective if the cost of genotyping decreases or if its diagnostic accuracy increases
Simons MJ, 2021	Decision tree model / State transition model	Simulated cohort (*n* = 1,000 per strategy)	GS as a diagnostic test + IHC to test for PD-L1 Standard of care followed by GS	Standard of care (NGS multi-gene panel, FISH, IHC, and Archer fusionPlex)	One-way Probabilistic One way and three ways threshold	GS is not cost effective compared with SoC. GS would be cost-effective if costs for GS decrease and additional patients with actionable targets are identified.
Simons MJ, 2023	Decision tree model/ State transition model	Simulated cohort (*n* = 1,000 per strategy)	GS as a diagnostic test + IHC to test for PD-L1 GS for treatment selection with novel targets GS-based biomarker for immunotherapy with novel targets Off-label drug approval based on GS results	Standard of care (NGS multi-gene panel, FISH, IHC, and Archer fusionPlex)	One-way Probabilistic	GS is not cost effective compared with SoC GS as a diagnostic test could become cost-effective if it detects more patients with actionable targets
Mfumbilwa ZA, 2024	Decision tree model/microsimulation model	Real-world Dutch patients with non-squamous metNSCLC (*n* = 2,196)*	GS as a diagnostic test for TMB alone GS as a diagnostic test for TMB + DB GS and IHC as a diagnostic test for TMB and PD-L1 GS and IHC as a diagnostic test for TMB and PD-L1 + DB	Standard of care (IHC, NGS and Archer fusionPLex; for PDL1) Standard of care (IHC, NGS and Archer fusionPLex) (for PDL1) + DB	One-way Probabilistic Threshold	GS is not cost effective compared with SoC. GS-TMB could become cost effective with a reduction in the cost of GS testing or an increase in the predictive value.
Fu J, 2025	Decision tree and partitioned survival models	Simulated cohort (*n* = 1,000)	GS as a diagnostic test to guide third line treatment selection	Standard of care (NGS testing for BRCA1/2 and/or IHC testing for dMMR) to guide third line treatment selection	One-way Probabilistic	GS is not cost effective compared to SoC GS would become cost-effective with a reduction in the costs of treatment and if it detects more patients with actionable targets
Diagnosis of rare genetic diseases						
Schofield D, 2019	Real world data + Counterfactual models	Real-world cohort (*n* = 80)	ES followed by outcomes in patients only (with and without reanalysis) outcomes in patients and first-degree relatives as a result of cascade testing outcomes in patients and first-degree relatives including parental reproductive outcomes	Standard of care (standard diagnostic care in infants with suspected monogenic disorders)	Bootstrap simulations	ES is increasingly cost-effective as the benefits of ES data reanalysis, cascade testing in first-degree relatives, and parental reproductive outcomes are taken into account
Stark Z, 2019	Real world data	Real-world cohort (*n* = 80, only 2 included in CUA)	Singleton ES as a first-tier sequencing test, followed by change in management in patient only changes in management in patient and first-degree relatives (cascade testing, and reproductive outcomes)	Standard of care (standard diagnostic care in infants with suspected monogenic disorders)	No	ES is cost-effective compared to SoC. (CUA on 2 patients only)
Crawford S, 2021	Decision tree model / Hybrid Markov model	Simulated cohorts of newborns in NICU (*n* = NS)	Trio eES Singleton eES	Trio TC Singleton TC	Decision tree model / Hybrid Markov model	Singleton and trio e(W)ES dominate current SoC
Avram CM, 2022	Decision analytic model	3 simulated cohorts of pregnant women with different gestational age (*n* = 470, *n* = 399, *n* = 430)	ES RASopathy panel followed by ES Metabolic panel followed by ES NIHF panel followed by ES	RASopathy panel Metabolic panel NIHF panel RASopathy panel followed by NIHF panel Metabolic panel followed by NIHF panel	One-way Multivariate	ES alone is the dominant strategy at all gestational ages
Lavelle TA, 2022	Decision tree model	2 simulated cohorts of infants/children (*n* = NS)	Standard of care followed by trio ES Standard of care followed by trio GS Standard of care followed by trio ES followed by trio GS First-line trio ES First-line trio GS Trio ES followed by GS (rapid trio for infants, standard trio for children)	Standard of care (single gene tests, panel tests, other laboratory tests)	One-way Probabilistic	Critically ill infants: GS is cost-effective. Non-critically ill children: GS is cost-effective under optimistic assumptions regarding their prognosis upon receiving a diagnosis
Sanford Kobayashi E, 2022	Comparison with counterfactual trajectories	Real-world cohort of children in PICU (*n* = 38)	rGS (Trio, duo and proband only)	Standard of care (counterfactual trajectories defined through a Delphi Consensus)	No	rGS is cost-effective in PICUs
Friedman MR, 2025	Markov decision model	Simulated cohort of pregnant women with low risk pregnancy (*n* = NS)	CMA + ES	CMA	One-way	ES + CMA has the potential to become cost-effective compared to CMA alone

*Mfumbilwa et al. (34). CMA, chromosomal microarray analysis; DB, disease burden; dMMR, mismatch repair-deficient; eES, early exome sequencing; FISH, fluorescence in situ hybridization; IHC, immunohistochemistry; NGS, next generation sequencing; PD-L1.

Simons et al. (32) found GS not cost-effective at baseline but potentially viable if sequencing costs decrease and additional biomarkers are identified. Their follow-up study (33) modeled three future scenarios, all proving cost-effective, especially when GS was used for treatment selection or immunotherapy biomarker identification. Mfumbilwa et al. (34) compared six strategies for immunotherapy selection, finding that finding that programmed death-ligand 1 (PD-L1) testing alone was the only cost-effective option (€74,900/QALY). However, the use of GS-based tumor mutational burden (TMB) could become cost-effective with lower testing costs or increased predictive value. Fu et al. (35) concluded that GS is currently not cost-effective for MRPC but could become viable if biomarker-guided therapy costs drop by 62 and 23% more patients with actionable targets are identified.

For treatment selection in patients with MDD, the strategy based solely on clinical risk factors was found to be more cost-effective than the one that combined clinical and genetic factors (ICER of £2,341 *vs* and £3,937 respectively). However, the latter strategy could become more cost-effective if the cost of genotyping decreases or if its diagnostic accuracy improves (31).

#### 3.4.2 Diagnosis of rare genetic diseases

GS/ES-based strategies were compared with conventional genetic tests for monogenic disorders, considering different sequencing approaches (singleton, duo, or trio; Table 4). Most studies (36–40) calculated ICER, while one (38) also calculated the iNMB using WTP thresholds of $50,000 and $200,000 per QALY. Sanford Kobayashi et al. (41) reported only cost per QALY gained.

Stark et al. (37) performed a CUA on two patients only, finding that ES was cost-saving when only clinical management changes were considered, but incurred additional costs when cascade testing and reproductive planning were included. The analysis by Schofield et al. (36) found that the cost-effectiveness of ES increases when data reanalysis, cascade testing in first-degree relatives, and parental reproductive outcomes are taken into account. Crawford et al. (38) found early ES (singleton and trio) cost-effective for newborns with suspected mitochondrial disorders, though scenario analyses highlighted limitations when ES was used as a late diagnostic tool. Avram et al. (39) compared 10 diagnostic pathways during pregnancy, finding ES the most cost-effective across all gestational ages. Lavelle et al. (40) evaluated ES and GS across seven testing strategies using a decision tree model with hypothetical cohorts of infants and children, showing that GS is cost-effective in diagnosing rare diseases for critically ill infants and possibly for non-critically ill children under optimistic cost assumptions. Sanford Kobayashi et al. (41) analyzed rapid GS (rGS) in a retrospective cohort, finding one-third of a QALY gained per patient at a fraction of typical cost-effectiveness thresholds, supporting rGS as a first-line test for selected cases. Friedman et al. (42) assessed ES combined with chromosomal microarray (CMA) for prenatal diagnosis in low-risk pregnancies, finding it cost-effective ($46,383/QALY), though effectiveness may decline when moderate/severe disabilities are detected.

## 4 Discussion

This systematic review investigated traditional full EEs of GS- and ES-based interventions that measured and integrated health outcomes, identifying CUAs conducted across five countries in the recent literature. Although cost-effectiveness results were reported, they were not the primary focus of the analysis; instead, we concentrated on how existing studies measure and incorporate health outcomes and where key methodological advances are still needed. Our findings reflect a substantial degree of methodological heterogeneity across the included studies: differences in time horizons, discount rates, analytical perspectives, and utility sources were frequent. Such inconsistencies limit the interpretability and generalizability of cost-effectiveness outcomes and illustrate the absence of standardized methodological expectations for economic evaluations of GS and ES.

We provided an overview of EE methods in two main clinical applications of GS and ES: treatment guidance and the diagnosis of genetic disorders.

In the first study area, research has mainly examined GS in oncology—especially advanced lung cancer (32–34), largely driven by a Dutch initiative (48)—with more recent applications in prostate cancer (35). These analyses generally showed strong methodological quality, especially in scenario definition and sensitivity analysis, which are essential for addressing uncertainty in early technology assessment (49). Across studies, sequencing costs, targeted therapy prices, and the probability of detecting actionable variants emerged as the main drivers of cost-effectiveness (32–35). The only study on psychiatric disorder (31) yielded similar results, suggesting that ES-guided treatment could become more cost-effective with lower treatment costs and improved diagnostic accuracy, while also highlighting pharmacogenomics as a promising area for future investigation. However, most treatment-guidance studies relied on simulated cohorts; only one used real-world data (34), limiting generalizability (50). In addition, GS costs were sometimes taken from commercial providers (32, 33), potentially over- or underestimating true costs. It should also be noted that GS/ES-guided treatment for cancer patients appeared potentially cost-effective when assessed against the Dutch willingness-to-pay threshold of €80,000 per QALY; however, this finding has limited generalizability, as differences in cost structures, treatment pathways, and WTP thresholds across healthcare systems may hinder its direct transferability to other settings., Additionally, due to data gaps, studies relied on assumptions about DY, uptake, treatment effectiveness, and patient response. For instance, Simons et al. estimated treatment effectiveness for rare variants due to the lack of randomized controlled trial data (33), a common challenge in precision medicine (51). This limitation could be addressed by emerging study designs, such as umbrella and basket trials, which enable a more efficient evaluation of targeted therapies in genetically defined subpopulations (52). Finally, uptake rates were often unreported, and some authors (34, 35) stressed the need to account for diagnostic waiting times, as these can influence treatment initiation and survival and should be incorporated to improve the realism of future evaluations (53). GS and ES are particularly valuable for the timely diagnosis of rare diseases, and our review suggests they may be cost-effective in this context; however evidence remains preliminary and context-dependent (36–42). Diagnostic strategies varied widely (e.g., rapid vs. standard sequencing, proband-only vs. trio sequencing), as did populations, clinical settings, and delivery models. Cost inclusion also differed markedly. While some studies included long-term care (38, 39), disability management (42), and reproductive services (36, 37), most lacked detailed downstream cost data and often excluded indirect costs, such as informal caregiving, productivity losses, and special education needs. Given that rare diseases frequently impose lifelong disability and family burden (54), failing to incorporate societal and informal costs may lead to systematic underestimation of true economic impact. Moreover, due to data scarcity, several studies relied on proxy conditions to estimate long-term outcomes and costs (38, 39). Methodological inconsistencies were common across diagnostic EEs, including differences in perspective, discount rates, time horizons, cohort size, and reporting transparency (37, 41, 42). To improve the comparability and reliability of future evaluations, the adoption of standardized, transparent reporting frameworks has been strongly recommended (55).

In estimating health benefits, most studies relied on common utility sources (43, 44) and validated multi-attribute utility instruments, including EQ-5D, HUI, and SF-6D (45–47). These instruments are also the most frequently recommended by HTA guidelines internationally (56, 57). In oncology and psychiatry, health states and disease progression were relatively well defined. In contrast, modeling outcomes for rare diseases proved more complex due to highly heterogeneous conditions and limited data on natural history. Here, health states often reflected levels of childhood disability and relied on caregiver-reported utilities, which are appropriate when patients cannot self-report their HRQOL (57). However, traditional utility measures remain limited in capturing the full value of genomic technologies. In particular, they struggle to incorporate non-health benefits, such as personal utility and the “value of knowing” (18, 19, 21, 58). There is growing methodological interest in expanding current frameworks by integrating patient preferences through discrete choice experiments (22), or connecting diagnostic yield with long-term survival and quality-of-life outcomes (20). Nonetheless, QALYs remain central to current HTA settings and are not fully replaceable (23, 59).

To strengthen future CUAs of genomic technologies, researchers should leverage updated utility data based on validated instruments, consider broader population-representative proxy conditions, and employ structured “impact inventories” to systematically map both health and non-health consequences (27).

Several genomics-specific challenges remain insufficiently addressed in current EEs, including test heterogeneity, patient stratification, incidental findings, data reanalysis, and spillover effects (19, 22, 60). While large genomic initiatives, such as population-based WGS studies (61), may help improve data availability for modeling, most evaluated studies did not yet incorporate these developments.

Notably, no study fully modeled the costs and benefits of incidental findings, despite their potential clinical, psychological, ethical and legal implications (22, 62–64). Similarly, data reanalysis—an increasingly important practice enabling reclassification of variants—was considered in only two studies (36, 37), even though it may substantially increase diagnostic yield over time (65, 66). Cascade testing and reproductive planning, which are particularly relevant in pediatric and familial genetic disorders, were also rarely included, despite their recognized importance (18).

Organizational and implementation aspects were almost entirely neglected. Only one study accounted for genetic counseling costs (36), and none fully evaluated workforce training, service delivery pathways, or infrastructure requirements. These omissions may lead to underestimation of real-world costs and limit the applicability of results for healthcare system planning. Even in the single study exploring sequencing as a population-level screening strategy (42), test delivery and organizational feasibility were not addressed.

To our knowledge, this is the first systematic review focused specifically on full economic evaluations of GS and ES that model health outcomes, with particular attention to how these outcomes are defined, quantified, and incorporated into economic models. This approach provides a structured methodological overview of current practices in a highly debated area of genomic medicine (67–69). Nevertheless, several limitations should be acknowledged. First, our primary objective was methodological rather than comparative; therefore, although we reported key parameters such as ICERs, willingness-to-pay thresholds, and iNMBs, we did not analyze these outcomes further. Second, by restricting inclusion to full EEs measuring health outcomes (i.e., QALYs and LYGs), and excluding other types of economic evaluations such as cost–benefit and cost–consequence analyses, we may have overlooked broader dimensions of value. Cost–benefit analyses can capture wider societal impacts, while cost–consequence analyses allow outcomes to be presented in a disaggregated manner. Third, limiting inclusion to English- and Italian-language publications may have introduced language bias and excluded relevant studies published in other languages; however, this was necessary to ensure accurate appraisal of complex methodological content. Finally, we did not conduct a meta-analysis due to substantial heterogeneity in study designs, cohort characteristics, and comparative strategies across the included studies.

In conclusion, although the value of GS and ES extends beyond clinical outcomes, current economic evaluations—particularly CUAs—struggle to capture this broader impact. Evidence on their cost-effectiveness in terms of QALYs remains limited and varies between treatment guidance and rare disease diagnosis. Future evaluations should incorporate long-term outcomes and real-world data, improve uncertainty handling, and better capture broader elements of value, including personal utility and organizational implications, to support informed and equitable decision-making.
