# Supplementary material for: Measuring the health benefits of genome and exome sequencing: a systematic review of economic evaluations
Source: Front Public Health. 2026 Jan 9;13:1728978. doi: 10.3389/fpubh.2025.1728978 (PMC12827763; doi:10.3389/fpubh.2025.1728978)
Supplement: Supplementary file 1 [file Data_Sheet_1.docx]

Supplementary Material

**Supplementary Table S1. Search strategies used in the systematic review for each database**

| **MEDLINE** |
| --- |
| ("genome sequencing” OR “genomic sequencing” OR “exome sequencing” OR “whole genome sequencing” OR “whole genomic sequencing” OR “whole exome sequencing” OR "next generation sequencing" OR "WGS" OR "WES" OR "NGS" OR "Whole Genome Sequencing"[Mesh] OR "Whole Exome Sequencing"[Mesh] OR "High-Throughput Nucleotide Sequencing"[Mesh]) AND ("economic evaluation" OR econom* OR cost* OR "cost-effectiveness" OR "cost-utility" OR "Costs and Cost Analysis"[Mesh]) |
| **Scopus** |
| TITLE-ABS-KEY(“genome sequencing” OR “genomic sequencing” OR “exome sequencing” OR “whole genome sequencing” OR “whole genomic sequencing” OR “whole exome sequencing” OR “next generation sequencing” OR “WGS” OR “WES” OR “NGS”) AND TITLE-ABS-KEY(“economic evaluation” OR econom* OR cost* OR “cost-effectiveness” OR “cost-utility”) |
| **Web of Science** |
| TS=(“genome sequencing” OR “genomic sequencing” OR “exome sequencing” OR “whole genome sequencing” OR “whole genomic sequencing” OR “whole exome sequencing” OR “next generation sequencing” OR “WGS” OR “WES” OR “NGS”) AND TS=(“economic evaluation” OR econom* OR cost* OR “cost-effectiveness” OR “cost-utility”) |
| **EconLit**  (“genome sequencing” OR “genomic sequencing” OR “exome sequencing” OR “whole genome sequencing” OR “whole genomic sequencing” OR “whole exome sequencing” OR “next generation sequencing” OR “WGS” OR “WES” OR “NGS”) AND (“economic evaluation” OR econom* OR cost* OR “cost-effectiveness” OR “cost-utility”) |
| **Centre for Reviews and Dissemination (CRD) University of York**  (sequenc* OR GS OR ES OR WGS OR WES OR NGS) AND (econ* OR effectiv* OR utilit*) |
| **Cost-effectiveness Analysis (CEA) Registry**  Keyword:"genome sequencing" OR "genomic sequencing" OR "exome sequencing" OR “whole genome sequencing” OR “whole genomic sequencing” OR “whole exome sequencing” OR "next generation sequencing" OR "WGS" OR "WES" OR "NGS" |
| **ICER Assessments Database**  (searching by single keyword) sequenc*;GS;ES;WGS; WES;NGS |

*We included “genome” and “exome” sequencing in the search strategies with and without the term “whole” in accordance with current genomic nomenclature recommendations (Jarvik GP, Evans JP. Mastering genomic terminology. Genet Med. 2017 May;19(5):491-492. doi: 10.1038/gim.2016.139. Epub 2016 Sep 22.)

**Supplementary Table S2. Sources of health outcomes modelled.**

| **Fist author, year** | **Measure and source of data** | | | **Reported references** |
| --- | --- | --- | --- | --- |
| **Targeted therapy guidance** | | | | |
| Fabbri C, 2020 | Utilities: cost-effectiveness studies (time trade-off and standard gamble) | | | Olgiati P, Bajo E, Bigelli M, De Ronchi D, Serretti A. Should pharmacogenetics be incorporated in major depression treatment? Economic evaluation in high- and middle-income European countries. Prog Neuropsychopharmacol Biol Psychiatry. 2012;36(1):147–154. doi: 10.1016/j.pnpbp.2011.08.013 |
|  |  |  |  | Hornberger J, Li Q, Quinn B. Cost-effectiveness of combinatorial pharmacogenomic testing for treatment-resistant major depressive disorder patients. Am J Manag Care. 2015;21(6):e357–e365. |
| Simons MJ, 2021 | Utilities: EQ-5D study | | | Chouaid C, Agulnik J, Goker E, Herder GJ, Lester JF, Vansteenkiste J, et al. Health-related quality of life and utility in patients with advanced non-small-cell lung cancer: a prospective cross-sectional patient survey in a real-world setting. J Thorac Oncol. 2013;8(8):997–1003. doi: 10.1097/JTO.0b013e318299243b. |
|  | Disutilities: standard gamble studies | | | Nafees B, Stafford M, Gavriel S, Bhalla S, Watkins J. Health state utilities for non-small cell lung cancer. Health Qual Life Outcomes. 2008 Oct 21;6:84. doi: 10.1186/1477-7525-6-84 |
|  |  |  |  | Lloyd A, Nafees B, Narewska J, Dewilde S, Watkins J. Health state utilities for metastatic breast cancer. Br J Cancer. 2006 Sep 18;95(6):683-90. doi: 10.1038/sj.bjc.6603326 |
|  | Disutilities: cost-effectiveness studies | | | Holleman MS, Al MJ, Zaim R, Groen HJM, Uyl-de Groot CA. Cost-effectiveness analysis of the first-line EGFR-TKIs in patients with non-small cell lung cancer harbouring EGFR mutations. Eur J Health Econ. 2020 Feb;21(1):153-164. doi: 10.1007/s10198-019-01117-3 |
|  |  |  |  | Westwood M, Joore M, Whiting P, van Asselt T, Ramaekers B, Armstrong N, Misso K, Severens J, Kleijnen J. Epidermal growth factor receptor tyrosine kinase (EGFR-TK) mutation testing in adults with locally advanced or metastatic non-small cell lung cancer: a systematic review and cost-effectiveness analysis. Health Technol Assess. 2014 May;18(32):1-166. doi: 10.3310/hta18320 |
| Simons MJ, 2023 | Utilities: EQ-5D study | | | Chouaid C, Agulnik J, Goker E, Herder GJ, Lester JF, Vansteenkiste J, et al. Health-related quality of life and utility in patients with advanced non-small-cell lung cancer: a prospective cross-sectional patient survey in a real-world setting. J Thorac Oncol. 2013;8(8):997–1003. doi: 10.1097/JTO.0b013e318299243b |
|  | Disutilities: standard gamble studies | | | Nafees B, Stafford M, Gavriel S, Bhalla S, Watkins J. Health state utilities for non-small cell lung cancer. Health Qual Life Outcomes. 2008 Oct 21;6:84. doi: 10.1186/1477-7525-6-84 |
|  |  |  |  | Lloyd A, Nafees B, Narewska J, Dewilde S, Watkins J. Health state utilities for metastatic breast cancer. Br J Cancer. 2006 Sep 18;95(6):683-90. doi: 10.1038/sj.bjc.6603326 |
|  | Disutilities: cost-effectiveness studies | | | Holleman MS, Al MJ, Zaim R, Groen HJM, Uyl-de Groot CA. Cost-effectiveness analysis of the first-line EGFR-TKIs in patients with non-small cell lung cancer harbouring EGFR mutations. Eur J Health Econ. 2020 Feb;21(1):153-164. doi: 10.1007/s10198-019-01117-3 |
|  |  |  |  | Westwood M, Joore M, Whiting P, van Asselt T, Ramaekers B, Armstrong N, Misso K, Severens J, Kleijnen J. Epidermal growth factor receptor tyrosine kinase (EGFR-TK) mutation testing in adults with locally advanced or metastatic non-small cell lung cancer: a systematic review and cost-effectiveness analysis. Health Technol Assess. 2014 May;18(32):1-166. doi: 10.3310/hta18320 |
| Mfumbilwa ZA, 2024 | Utilities: EQ-5D study | | | Chouaid C, Agulnik J, Goker E, Herder GJ, Lester JF, Vansteenkiste J, et al. Health-related quality of life and utility in patients with advanced non-small-cell lung cancer: a prospective cross-sectional patient survey in a real-world setting. J Thorac Oncol. 2013;8(8):997–1003. doi: 10.1097/JTO.0b013e318299243b |
|  | Disutilities: standard gamble studies and time-trade off studies | | | Nafees B, Stafford M, Gavriel S, Bhalla S, Watkins J. Health state utilities for non-small cell lung cancer. Health Qual Life Outcomes. 2008 Oct 21;6:84. doi: 10.1186/1477-7525-6-84 |
|  |  |  |  | Paracha N, Abdulla A, MacGilchrist KS. Systematic review of health state utility values in metastatic non-small cell lung cancer with a focus on previously treated patients. Health Qual Life Outcomes. 2018 Sep 12;16(1):179. doi: 10.1186/s12955-018-0994-8 |
|  |  |  |  | Lloyd A, Nafees B, Narewska J, Dewilde S, Watkins J. Health state utilities for metastatic breast cancer. Br J Cancer. 2006 Sep 18;95(6):683-90. doi: 10.1038/sj.bjc.6603326 |
|  | Disutilities: cost-effectiveness studies | | | Holleman MS, Al MJ, Zaim R, Groen HJM, Uyl-de Groot CA. Cost-effectiveness analysis of the first-line EGFR-TKIs in patients with non-small cell lung cancer harbouring EGFR mutations. Eur J Health Econ. 2020 Feb;21(1):153-164. doi: 10.1007/s10198-019-01117-3 |
| Fu J 2025 | Utilities: EQ-5D study | | | Torvinen S, Färkkilä N, Sintonen H, Saarto T, Roine RP, Taari K. Health-related quality of life in prostate cancer. *Acta Oncologica*. 2013-08-01 2013;52(6):1094-1101. doi:10.3109/0284186x.2012.760848 |
|  | Disutilities; time-trade off study | | | Matza LS, Cong Z, Chung K, et al. Utilities associated with subcutaneous injections and intravenous infusions for treatment of patients with bone metastases. Patient Prefer Adherence. 2013;7:855-65. doi:10.2147/PPA.S44947 |
|  | Disutilities; trial data | | | De Wit R, De Bono J, Sternberg CN, et al. Cabazitaxel versus Abiraterone or Enzalutamide in Metastatic Prostate Cancer. New England Journal of Medicine. 2019-12-26 2019;381(26):2506-2518. doi:10.1056/nejmoa1911206 |
|  |  |  |  | Maio M, Ascierto PA, Manzyuk L, et al. Pembrolizumab in microsatellite instability high or mismatch repair deficient cancers: updated analysis from the phase II KEYNOTE-158 study. Annals of Oncology. 2022-09-01 2022;33(9):929-938. doi: 10.1016/j.annonc.2022.05.519 |
|  |  |  |  | Marabelle A, Fakih M, Lopez J, et al. Association of tumour mutational burden with outcomes in patients with advanced solid tumours treated with pembrolizumab: prospective biomarker analysis of the multicohort, open-label, phase 2 KEYNOTE-158 study. Lancet Oncol. Oct 2020;21(10):1353-1365. doi:10.1016/S1470-2045(20)30445-9 |
|  |  |  |  | de Bono J, Mateo J, Fizazi K, et al. Olaparib for Metastatic Castration-Resistant Prostate Cancer. N Engl J Med. May 28 2020;382(22):2091-2102. doi:10.1056/NEJMoa1911440 |
| **Diagnosis of rare genetic diseases** | | | | |
| Schofield D, 2019 | | Utilities- parents: parental  preferences survey  (standard gamble and time trade-off) | Carroll AE, Downs SM. Improving decision analyses: parent preferences (utility values) for pediatric health outcomes. J Pediatr. 2009;155(1):21- 25, 25.e1-25.e5 doi:10.1016/j.jpeds.2009.01.040 | |
|  |  | Utilities- bone marrow transplantation: meta-analysis based on HUI-2 | Kwon J, Kim SW, Ungar WJ, Tsiplova K, Madan J, Petrou S. A Systematic Review and Meta-analysis of Childhood Health Utilities. Med Decis Making. 2018 Apr;38(3):277-305. doi: 10.1177/0272989X17732990 | |
|  |  | Utilities - fertility: HUI-2 | Torrance GW, Feeny DH, Furlong WJ, Barr RD, Zhang Y, Wang Q. Multiattribute utility function for a comprehensive health status classification system. Health Utilities Index Mark 2. Med Care. 1996 Jul;34(7):702-22. doi: 10.1097/00005650-199607000-00004 | |
| Stark Z, 2019 | | Utilities- parents: parental  preferences survey  (standard gamble and time trade-off) | Carroll AE, Downs SM. Improving decision analyses: parent preferences (utility values) for pediatric health outcomes. J Pediatr. 2009;155(1):21- 25, 25.e1-25.e5 https://doi.org/10.1016/j.jpeds.2009.01.040 | |
|  |  | Utilities - fertility: cost-effectiveness study based on HUI-2 | Scotland GS, McLernon D, Kurinczuk JJ, McNamee P, Harrild K, Lyall H, Rajkhowa M, Hamilton M, Bhattacharya S. Minimising twins in in vitro fertilisation: a modelling study assessing the costs, consequences and cost-utility of elective single versus double embryo transfer over a 20-year time horizon. BJOG. 2011 Aug;118(9):1073-83. doi: 10.1111/j.1471-0528.2011.02966.x | |
| Crawford S, 2021 | | Utilities- parents: parental  preferences survey  (standard gamble and time trade-off) | Carroll AE, Downs SM. Improving decision analyses: parent preferences (utility values) for pediatric health outcomes. J Pediatr. 2009;155(1):21- 25, 25.e1-25.e5 doi:10.1016/j.jpeds.2009.01.040 | |
|  |  | Utilities- childhood conditions: HUI-3 | Petrou S, Kupek E. Estimating preference-based health utilities index mark 3 utility scores for childhood conditions in England and Scotland. Med Decis Making. 2009 May-Jun;29(3):291-303. doi: 10.1177/0272989X08327398 | |
| Avram CM, 2022 | | Utilities- mothers: cost-effectiveness study based on time trade-off data | Petrou S, Kupek E. Estimating preference-based health utilities index mark 3 utility scores for childhood conditions in England and Scotland. Med Decis Making. 2009 May-Jun;29(3):291-303. doi: 10.1177/0272989X08327398 | |
|  |  | Utilities-mothers: survey on Down syndrome (standard gamble) | Grobman WA, Dooley SL, Welshman EE, Pergament E, Calhoun EA. Preference assessment of prenatal diagnosis for Down syndrome: is 35 years a rational cutoff? Prenat Diagn. 2002 Dec;22(13):1195-200. doi: 10.1002/pd.494 | |
|  |  | Utilities- parents: study on preferences for neonatal outcomes  (standard gamble) | Saigal S, Stoskopf BL, Feeny D, Furlong W, Burrows E, Rosenbaum PL, Hoult L. Differences in preferences for neonatal outcomes among health care professionals, parents, and adolescents. JAMA. 1999 Jun 2;281(21):1991-7. doi: 10.1001/jama.281.21.1991 | |
|  |  | Utilities-parents: cost-effectiveness study based on time-trade off data | Harris RA, Washington AE, Nease RF Jr, Kuppermann M. Cost utility of prenatal diagnosis and the risk-based threshold. Lancet. 2004 Jan 24;363(9405):276-82. doi: 10.1016/S0140-6736(03)15385-8 | |
| Lavelle TA, 2022 | | Utilities- parents: parental  preferences survey  (standard gamble and time trade-off) | Carroll AE, Downs SM. Improving decision analyses: parent preferences (utility values) for pediatric health outcomes. J Pediatr. 2009;155(1):21- 25, 25.e1-25.e5 https://doi.org/10.1016/j.jpeds.2009.01.040. | |
|  |  | Utilities: SF-6D study | Ara R, Brazier J. Estimating health state utility values for comorbid health conditions using SF-6D data. Value Health. 2011 Jul-Aug;14(5):740-5. doi: 10.1016/j.jval.2010.12.011 | |
| Sanford Kobayashi E, 2022 | | Delphi panel | NA | |
| Friedman MR, 2025 | | Not specified | NA | |

NA: Not applicable

**Supplementary Data. Quality evaluation of included studies**

**The Quality of Health Economic Studies (QHES) instrument -** *Fabbri, 2020*

| **No** | **Questions** | **Points** | **Yes** | **No** |
| --- | --- | --- | --- | --- |
| 1. | Was the study objective presented in a clear, specific, and measurable manner? | 7 | x |  |
| 2. | Were the perspective of the analysis (societal, third-party payer, etc.) and reasons for its selection stated? | 4 | x |  |
| 3. | Were variable estimates used in the analysis from the best available source (i.e., randomized control trial - best, expert opinion - worst)? | 8 | x |  |
| 4. | If estimates came from a subgroup analysis, were the groups pre- specified at the beginning of the study? | 1 | x (NA) |  |
| 5. | Was uncertainty handled by (1) statistical analysis to address random events, (2) sensitivity analysis to cover a range of assumptions? | 9 | x |  |
| 6. | Was incremental analysis performed between alternatives for resources and costs? | 6 | x |  |
| 7. | Was the methodology for data abstraction (including the value of health states and other benefits) stated? | 5 | x |  |
| 8. | Did the analytic horizon allow time for all relevant and important outcomes? Were benefits and costs that went beyond 1 year discounted (3% to 5%) and justification given for the discount rate? | 7 |  | x |
| 9. | Was the measurement of costs appropriate and the methodology for the estimation of quantities and unit costs clearly described? | 8 | x |  |
| 10. | Were the primary outcome measure(s) for the economic evaluation  clearly stated and did they include the major short-term, long-term, and negative outcomes? | 6 | x |  |
| 11. | Were the health outcomes measures/scales valid and reliable? If previously tested valid and reliable measures were not available, was justification given for the measures/scales used? | 7 | x |  |
| 12. | Were the economic model (including structure), study methods and analysis, and the components of the numerator and denominator displayed in a clear, transparent manner? | 8 | x |  |
| 13. | Were the choice of economic model, main assumptions, and limitations of the study stated and justified? | 7 | x |  |
| 14. | Did the author(s) explicitly discuss direction and magnitude of potential biases? | 6 | x |  |
| 15. | Were the conclusions/recommendations of the study justified and based on the study results? | 8 | x |  |
| 16. | Was there a statement disclosing the source of funding for the study? | 3 | x |  |
|  | **TOTAL POINTS** | **100** | 93 |  |

**The Quality of Health Economic Studies (QHES) instrument -** *Simons, 2021*

| **No** | **Questions** | **Points** | **Yes** | **No** |
| --- | --- | --- | --- | --- |
| 1. | Was the study objective presented in a clear, specific, and measurable manner? | 7 | x |  |
| 2. | Were the perspective of the analysis (societal, third-party payer, etc.) and reasons for its selection stated? | 4 | x |  |
| 3. | Were variable estimates used in the analysis from the best available source (i.e., randomized control trial - best, expert opinion - worst)? | 8 | x |  |
| 4. | If estimates came from a subgroup analysis, were the groups pre- specified at the beginning of the study? | 1 | x |  |
| 5. | Was uncertainty handled by (1) statistical analysis to address random events, (2) sensitivity analysis to cover a range of assumptions? | 9 | x |  |
| 6. | Was incremental analysis performed between alternatives for resources and costs? | 6 | x |  |
| 7. | Was the methodology for data abstraction (including the value of health states and other benefits) stated? | 5 | x |  |
| 8. | Did the analytic horizon allow time for all relevant and important outcomes? Were benefits and costs that went beyond 1 year discounted (3% to 5%) and justification given for the discount rate? | 7 | x |  |
| 9. | Was the measurement of costs appropriate and the methodology for the estimation of quantities and unit costs clearly described? | 8 | x |  |
| 10. | Were the primary outcome measure(s) for the economic evaluation  clearly stated and did they include the major short-term, long-term, and negative outcomes? | 6 | x |  |
| 11. | Were the health outcomes measures/scales valid and reliable? If previously tested valid and reliable measures were not available, was justification given for the measures/scales used? | 7 | x |  |
| 12. | Were the economic model (including structure), study methods and analysis, and the components of the numerator and denominator displayed in a clear, transparent manner? | 8 | x |  |
| 13. | Were the choice of economic model, main assumptions, and limitations of the study stated and justified? | 7 | x |  |
| 14. | Did the author(s) explicitly discuss direction and magnitude of potential biases? | 6 | x |  |
| 15. | Were the conclusions/recommendations of the study justified and based on the study results? | 8 | x |  |
| 16. | Was there a statement disclosing the source of funding for the study? | 3 | x |  |
|  | **TOTAL POINTS** | **100** | 100 |  |

| **No** | **Questions** | **Points** | **Yes** | **No** |
| --- | --- | --- | --- | --- |
| 1. | Was the study objective presented in a clear, specific, and measurable manner? | 7 | x |  |
| 2. | Were the perspective of the analysis (societal, third-party payer, etc.) and reasons for its selection stated? | 4 | x |  |
| 3. | Were variable estimates used in the analysis from the best available source (i.e., randomized control trial - best, expert opinion - worst)? | 8 | x |  |
| 4. | If estimates came from a subgroup analysis, were the groups pre- specified at the beginning of the study? | 1 | x  (NA) |  |
| 5. | Was uncertainty handled by (1) statistical analysis to address random events, (2) sensitivity analysis to cover a range of assumptions? | 9 | x |  |
| 6. | Was incremental analysis performed between alternatives for resources and costs? | 6 | x |  |
| 7. | Was the methodology for data abstraction (including the value of health states and other benefits) stated? | 5 | x |  |
| 8. | Did the analytic horizon allow time for all relevant and important outcomes? Were benefits and costs that went beyond 1 year discounted (3% to 5%) and justification given for the discount rate? | 7 | x |  |
| 9. | Was the measurement of costs appropriate and the methodology for the estimation of quantities and unit costs clearly described? | 8 | x |  |
| 10. | Were the primary outcome measure(s) for the economic evaluation  clearly stated and did they include the major short-term, long-term, and negative outcomes? | 6 |  | x |
| 11. | Were the health outcomes measures/scales valid and reliable? If previously tested valid and reliable measures were not available, was justification given for the measures/scales used? | 7 | x |  |
| 12. | Were the economic model (including structure), study methods and analysis, and the components of the numerator and denominator displayed in a clear, transparent manner? | 8 | x |  |
| 13. | Were the choice of economic model, main assumptions, and limitations of the study stated and justified? | 7 | x |  |
| 14. | Did the author(s) explicitly discuss direction and magnitude of potential biases? | 6 | x |  |
| 15. | Were the conclusions/recommendations of the study justified and based on the study results? | 8 | x |  |
| 16. | Was there a statement disclosing the source of funding for the study? | 3 | x |  |
|  | **TOTAL POINTS** | **100** | 94 |  |

**The Quality of Health Economic Studies (QHES) instrument** *- Simons, 2023*

**The Quality of Health Economic Studies (QHES) instrument -** *Mfumbilwa, 2024*

| **No** | **Questions** | **Points** | **Yes** | **No** |
| --- | --- | --- | --- | --- |
| 1. | Was the study objective presented in a clear, specific, and measurable manner? | 7 | x |  |
| 2. | Were the perspective of the analysis (societal, third-party payer, etc.) and reasons for its selection stated? | 4 | x |  |
| 3. | Were variable estimates used in the analysis from the best available source (i.e., randomized control trial - best, expert opinion - worst)? | 8 | x |  |
| 4. | If estimates came from a subgroup analysis, were the groups pre- specified at the beginning of the study? | 1 | x |  |
| 5. | Was uncertainty handled by (1) statistical analysis to address random events, (2) sensitivity analysis to cover a range of assumptions? | 9 | x |  |
| 6. | Was incremental analysis performed between alternatives for resources and costs? | 6 | x |  |
| 7. | Was the methodology for data abstraction (including the value of health states and other benefits) stated? | 5 | x |  |
| 8. | Did the analytic horizon allow time for all relevant and important outcomes? Were benefits and costs that went beyond 1 year discounted (3% to 5%) and justification given for the discount rate? | 7 | x |  |
| 9. | Was the measurement of costs appropriate and the methodology for the estimation of quantities and unit costs clearly described? | 8 | x |  |
| 10. | Were the primary outcome measure(s) for the economic evaluation  clearly stated and did they include the major short-term, long-term, and negative outcomes? | 6 | x |  |
| 11. | Were the health outcomes measures/scales valid and reliable? If previously tested valid and reliable measures were not available, was justification given for the measures/scales used? | 7 | x |  |
| 12. | Were the economic model (including structure), study methods and analysis, and the components of the numerator and denominator displayed in a clear, transparent manner? | 8 |  | x |
| 13. | Were the choice of economic model, main assumptions, and limitations of the study stated and justified? | 7 | x |  |
| 14. | Did the author(s) explicitly discuss direction and magnitude of potential biases? | 6 | x |  |
| 15. | Were the conclusions/recommendations of the study justified and based on the study results? | 8 | x |  |
| 16. | Was there a statement disclosing the source of funding for the study? | 3 | x |  |
|  | **TOTAL POINTS** | **100** | 92 |  |

**The Quality of Health Economic Studies (QHES) instrument -** *Fu, 2025*

| **No** | **Questions** | **Points** | **Yes** | **No** |
| --- | --- | --- | --- | --- |
| 1. | Was the study objective presented in a clear, specific, and measurable manner? | 7 | x |  |
| 2. | Were the perspective of the analysis (societal, third-party payer, etc.) and reasons for its selection stated? | 4 | x |  |
| 3. | Were variable estimates used in the analysis from the best available source (i.e., randomized control trial - best, expert opinion - worst)? | 8 | x |  |
| 4. | If estimates came from a subgroup analysis, were the groups pre- specified at the beginning of the study? | 1 | x |  |
| 5. | Was uncertainty handled by (1) statistical analysis to address random events, (2) sensitivity analysis to cover a range of assumptions? | 9 | x |  |
| 6. | Was incremental analysis performed between alternatives for resources and costs? | 6 | x |  |
| 7. | Was the methodology for data abstraction (including the value of health states and other benefits) stated? | 5 | x |  |
| 8. | Did the analytic horizon allow time for all relevant and important outcomes? Were benefits and costs that went beyond 1 year discounted (3% to 5%) and justification given for the discount rate? | 7 | x |  |
| 9. | Was the measurement of costs appropriate and the methodology for the estimation of quantities and unit costs clearly described? | 8 | x |  |
| 10. | Were the primary outcome measure(s) for the economic evaluation  clearly stated and did they include the major short-term, long-term, and negative outcomes? | 6 | x |  |
| 11. | Were the health outcomes measures/scales valid and reliable? If previously tested valid and reliable measures were not available, was justification given for the measures/scales used? | 7 | x |  |
| 12. | Were the economic model (including structure), study methods and analysis, and the components of the numerator and denominator displayed in a clear, transparent manner? | 8 | x |  |
| 13. | Were the choice of economic model, main assumptions, and limitations of the study stated and justified? | 7 | x |  |
| 14. | Did the author(s) explicitly discuss direction and magnitude of potential biases? | 6 | x |  |
| 15. | Were the conclusions/recommendations of the study justified and based on the study results? | 8 | x |  |
| 16. | Was there a statement disclosing the source of funding for the study? | 3 | x |  |
|  | **TOTAL POINTS** | **100** | 100 |  |

**The Quality of Health Economic Studies (QHES) instrument -** *Schofield, 2019*

| **No** | **Questions** | **Points** | **Yes** | **No** |
| --- | --- | --- | --- | --- |
| 1. | Was the study objective presented in a clear, specific, and measurable manner? | 7 | x |  |
| 2. | Were the perspective of the analysis (societal, third-party payer, etc.) and reasons for its selection stated? | 4 | x |  |
| 3. | Were variable estimates used in the analysis from the best available source (i.e., randomized control trial - best, expert opinion - worst)? | 8 | x |  |
| 4. | If estimates came from a subgroup analysis, were the groups pre- specified at the beginning of the study? | 1 | x  (NA) |  |
| 5. | Was uncertainty handled by (1) statistical analysis to address random events, (2) sensitivity analysis to cover a range of assumptions? | 9 | x |  |
| 6. | Was incremental analysis performed between alternatives for resources and costs? | 6 | x |  |
| 7. | Was the methodology for data abstraction (including the value of health states and other benefits) stated? | 5 | x |  |
| 8. | Did the analytic horizon allow time for all relevant and important outcomes? Were benefits and costs that went beyond 1 year discounted (3% to 5%) and justification given for the discount rate? | 7 |  | x |
| 9. | Was the measurement of costs appropriate and the methodology for the estimation of quantities and unit costs clearly described? | 8 | x |  |
| 10. | Were the primary outcome measure(s) for the economic evaluation  clearly stated and did they include the major short-term, long-term, and negative outcomes? | 6 |  | x |
| 11. | Were the health outcomes measures/scales valid and reliable? If previously tested valid and reliable measures were not available, was justification given for the measures/scales used? | 7 | x |  |
| 12. | Were the economic model (including structure), study methods and analysis, and the components of the numerator and denominator displayed in a clear, transparent manner? | 8 | x |  |
| 13. | Were the choice of economic model, main assumptions, and limitations of the study stated and justified? | 7 | x |  |
| 14. | Did the author(s) explicitly discuss direction and magnitude of potential biases? | 6 | x |  |
| 15. | Were the conclusions/recommendations of the study justified and based on the study results? | 8 | x |  |
| 16. | Was there a statement disclosing the source of funding for the study? | 3 |  | x |
|  | **TOTAL POINTS** | **100** | 84 |  |

**The Quality of Health Economic Studies (QHES) instrument -** *Stark, 2019*

| **No** | **Questions** | **Points** | **Yes** | **No** |
| --- | --- | --- | --- | --- |
| 1. | Was the study objective presented in a clear, specific, and measurable manner? | 7 | x |  |
| 2. | Were the perspective of the analysis (societal, third-party payer, etc.) and reasons for its selection stated? | 4 | x |  |
| 3. | Were variable estimates used in the analysis from the best available source (i.e., randomized control trial - best, expert opinion - worst)? | 8 | x |  |
| 4. | If estimates came from a subgroup analysis, were the groups pre- specified at the beginning of the study? | 1 | x  (NA) |  |
| 5. | Was uncertainty handled by (1) statistical analysis to address random events, (2) sensitivity analysis to cover a range of assumptions? | 9 |  | x |
| 6. | Was incremental analysis performed between alternatives for resources and costs? | 6 | x |  |
| 7. | Was the methodology for data abstraction (including the value of health states and other benefits) stated? | 5 | x |  |
| 8. | Did the analytic horizon allow time for all relevant and important outcomes? Were benefits and costs that went beyond 1 year discounted (3% to 5%) and justification given for the discount rate? | 7 |  | x |
| 9. | Was the measurement of costs appropriate and the methodology for the estimation of quantities and unit costs clearly described? | 8 |  | x |
| 10. | Were the primary outcome measure(s) for the economic evaluation  clearly stated and did they include the major short-term, long-term, and negative outcomes? | 6 |  | x |
| 11. | Were the health outcomes measures/scales valid and reliable? If previously tested valid and reliable measures were not available, was justification given for the measures/scales used? | 7 | x |  |
| 12. | Were the economic model (including structure), study methods and analysis, and the components of the numerator and denominator displayed in a clear, transparent manner? | 8 | x |  |
| 13. | Were the choice of economic model, main assumptions, and limitations of the study stated and justified? | 7 | x |  |
| 14. | Did the author(s) explicitly discuss direction and magnitude of potential biases? | 6 | x |  |
| 15. | Were the conclusions/recommendations of the study justified and based on the study results? | 8 | x |  |
| 16. | Was there a statement disclosing the source of funding for the study? | 3 | x |  |
|  | **TOTAL POINTS** | **100** | 70 |  |

**The Quality of Health Economic Studies (QHES) instrument -** *Crawford, 2021*

| **No** | **Questions** | **Points** | **Yes** | **No** |
| --- | --- | --- | --- | --- |
| 1. | Was the study objective presented in a clear, specific, and measurable manner? | 7 | x |  |
| 2. | Were the perspective of the analysis (societal, third-party payer, etc.) and reasons for its selection stated? | 4 | x |  |
| 3. | Were variable estimates used in the analysis from the best available source (i.e., randomized control trial - best, expert opinion - worst)? | 8 | x |  |
| 4. | If estimates came from a subgroup analysis, were the groups pre- specified at the beginning of the study? | 1 | x  (NA) |  |
| 5. | Was uncertainty handled by (1) statistical analysis to address random events, (2) sensitivity analysis to cover a range of assumptions? | 9 | x |  |
| 6. | Was incremental analysis performed between alternatives for resources and costs? | 6 | x |  |
| 7. | Was the methodology for data abstraction (including the value of health states and other benefits) stated? | 5 | x |  |
| 8. | Did the analytic horizon allow time for all relevant and important outcomes? Were benefits and costs that went beyond 1 year discounted (3% to 5%) and justification given for the discount rate? | 7 | x |  |
| 9. | Was the measurement of costs appropriate and the methodology for the estimation of quantities and unit costs clearly described? | 8 | x |  |
| 10. | Were the primary outcome measure(s) for the economic evaluation  clearly stated and did they include the major short-term, long-term, and negative outcomes? | 6 |  | x |
| 11. | Were the health outcomes measures/scales valid and reliable? If previously tested valid and reliable measures were not available, was justification given for the measures/scales used? | 7 | x |  |
| 12. | Were the economic model (including structure), study methods and analysis, and the components of the numerator and denominator displayed in a clear, transparent manner? | 8 | x |  |
| 13. | Were the choice of economic model, main assumptions, and limitations of the study stated and justified? | 7 | x |  |
| 14. | Did the author(s) explicitly discuss direction and magnitude of potential biases? | 6 | x |  |
| 15. | Were the conclusions/recommendations of the study justified and based on the study results? | 8 | x |  |
| 16. | Was there a statement disclosing the source of funding for the study? | 3 |  | x |
|  | **TOTAL POINTS** | **100** | 91 |  |

**The Quality of Health Economic Studies (QHES) instrument -** *Avram, 2022*

| **No** | **Questions** | **Points** | **Yes** | **No** |
| --- | --- | --- | --- | --- |
| 1. | Was the study objective presented in a clear, specific, and measurable manner? | 7 | x |  |
| 2. | Were the perspective of the analysis (societal, third-party payer, etc.) and reasons for its selection stated? | 4 | x |  |
| 3. | Were variable estimates used in the analysis from the best available source (i.e., randomized control trial - best, expert opinion - worst)? | 8 | x |  |
| 4. | If estimates came from a subgroup analysis, were the groups pre- specified at the beginning of the study? | 1 | x |  |
| 5. | Was uncertainty handled by (1) statistical analysis to address random events, (2) sensitivity analysis to cover a range of assumptions? | 9 | x |  |
| 6. | Was incremental analysis performed between alternatives for resources and costs? | 6 | x |  |
| 7. | Was the methodology for data abstraction (including the value of health states and other benefits) stated? | 5 | x |  |
| 8. | Did the analytic horizon allow time for all relevant and important outcomes? Were benefits and costs that went beyond 1 year discounted (3% to 5%) and justification given for the discount rate? | 7 |  | x |
| 9. | Was the measurement of costs appropriate and the methodology for the estimation of quantities and unit costs clearly described? | 8 | x |  |
| 10. | Were the primary outcome measure(s) for the economic evaluation  clearly stated and did they include the major short-term, long-term, and negative outcomes? | 6 | x |  |
| 11. | Were the health outcomes measures/scales valid and reliable? If previously tested valid and reliable measures were not available, was justification given for the measures/scales used? | 7 | x |  |
| 12. | Were the economic model (including structure), study methods and analysis, and the components of the numerator and denominator displayed in a clear, transparent manner? | 8 | x |  |
| 13. | Were the choice of economic model, main assumptions, and limitations of the study stated and justified? | 7 | x |  |
| 14. | Did the author(s) explicitly discuss direction and magnitude of potential biases? | 6 | x |  |
| 15. | Were the conclusions/recommendations of the study justified and based on the study results? | 8 | x |  |
| 16. | Was there a statement disclosing the source of funding for the study? | 3 | x |  |
|  | **TOTAL POINTS** | **100** | 93 |  |

**The Quality of Health Economic Studies (QHES) instrument -** *Lavelle, 2022*

| **No** | **Questions** | **Points** | **Yes** | **No** |
| --- | --- | --- | --- | --- |
| 1. | Was the study objective presented in a clear, specific, and measurable manner? | 7 | x |  |
| 2. | Were the perspective of the analysis (societal, third-party payer, etc.) and reasons for its selection stated? | 4 | x |  |
| 3. | Were variable estimates used in the analysis from the best available source (i.e., randomized control trial - best, expert opinion - worst)? | 8 | x |  |
| 4. | If estimates came from a subgroup analysis, were the groups pre- specified at the beginning of the study? | 1 | x |  |
| 5. | Was uncertainty handled by (1) statistical analysis to address random events, (2) sensitivity analysis to cover a range of assumptions? | 9 | x |  |
| 6. | Was incremental analysis performed between alternatives for resources and costs? | 6 | x |  |
| 7. | Was the methodology for data abstraction (including the value of health states and other benefits) stated? | 5 | x |  |
| 8. | Did the analytic horizon allow time for all relevant and important outcomes? Were benefits and costs that went beyond 1 year discounted (3% to 5%) and justification given for the discount rate? | 7 | x |  |
| 9. | Was the measurement of costs appropriate and the methodology for the estimation of quantities and unit costs clearly described? | 8 | x |  |
| 10. | Were the primary outcome measure(s) for the economic evaluation  clearly stated and did they include the major short-term, long-term, and negative outcomes? | 6 |  | x |
| 11. | Were the health outcomes measures/scales valid and reliable? If previously tested valid and reliable measures were not available, was justification given for the measures/scales used? | 7 | x |  |
| 12. | Were the economic model (including structure), study methods and analysis, and the components of the numerator and denominator displayed in a clear, transparent manner? | 8 | x |  |
| 13. | Were the choice of economic model, main assumptions, and limitations of the study stated and justified? | 7 | x |  |
| 14. | Did the author(s) explicitly discuss direction and magnitude of potential biases? | 6 | x |  |
| 15. | Were the conclusions/recommendations of the study justified and based on the study results? | 8 | x |  |
| 16. | Was there a statement disclosing the source of funding for the study? | 3 | x |  |
|  | **TOTAL POINTS** | **100** | 94 |  |

**The Quality of Health Economic Studies (QHES) instrument -** *Sanford* *Kobayashi, 2022*

| **No** | **Questions** | **Points** | **Yes** | **No** |
| --- | --- | --- | --- | --- |
| 1. | Was the study objective presented in a clear, specific, and measurable manner? | 7 | x |  |
| 2. | Were the perspective of the analysis (societal, third-party payer, etc.) and reasons for its selection stated? | 4 |  | x |
| 3. | Were variable estimates used in the analysis from the best available source (i.e., randomized control trial - best, expert opinion - worst)? | 8 | x |  |
| 4. | If estimates came from a subgroup analysis, were the groups pre- specified at the beginning of the study? | 1 | x  (NA) |  |
| 5. | Was uncertainty handled by (1) statistical analysis to address random events, (2) sensitivity analysis to cover a range of assumptions? | 9 |  | x |
| 6. | Was incremental analysis performed between alternatives for resources and costs? | 6 |  | x |
| 7. | Was the methodology for data abstraction (including the value of health states and other benefits) stated? | 5 | x |  |
| 8. | Did the analytic horizon allow time for all relevant and important outcomes? Were benefits and costs that went beyond 1 year discounted (3% to 5%) and justification given for the discount rate? | 7 |  | x |
| 9. | Was the measurement of costs appropriate and the methodology for the estimation of quantities and unit costs clearly described? | 8 | x |  |
| 10. | Were the primary outcome measure(s) for the economic evaluation  clearly stated and did they include the major short-term, long-term, and negative outcomes? | 6 |  | x |
| 11. | Were the health outcomes measures/scales valid and reliable? If previously tested valid and reliable measures were not available, was justification given for the measures/scales used? | 7 | x |  |
| 12. | Were the economic model (including structure), study methods and analysis, and the components of the numerator and denominator displayed in a clear, transparent manner? | 8 |  | x |
| 13. | Were the choice of economic model, main assumptions, and limitations of the study stated and justified? | 7 | x |  |
| 14. | Did the author(s) explicitly discuss direction and magnitude of potential biases? | 6 | x |  |
| 15. | Were the conclusions/recommendations of the study justified and based on the study results? | 8 | x |  |
| 16. | Was there a statement disclosing the source of funding for the study? | 3 | x |  |
|  | **TOTAL POINTS** | **100** | 60 |  |

**The Quality of Health Economic Studies (QHES) instrument -** *Rosenberg Friedman, 2025*

| **No** | **Questions** | **Points** | **Yes** | **No** |
| --- | --- | --- | --- | --- |
| 1. | Was the study objective presented in a clear, specific, and measurable manner? | 1 | x |  |
| 2. | Were the perspective of the analysis (societal, third-party payer, etc.) and reasons for its selection stated? | 4 |  | x |
| 3. | Were variable estimates used in the analysis from the best available source (i.e., randomized control trial - best, expert opinion - worst)? | 8 |  | x |
| 4. | If estimates came from a subgroup analysis, were the groups pre- specified at the beginning of the study? | 1 | x  (NA) |  |
| 5. | Was uncertainty handled by (1) statistical analysis to address random events, (2) sensitivity analysis to cover a range of assumptions? | 9 | x |  |
| 6. | Was incremental analysis performed between alternatives for resources and costs? | 6 | x |  |
| 7. | Was the methodology for data abstraction (including the value of health states and other benefits) stated? | 5 |  | x |
| 8. | Did the analytic horizon allow time for all relevant and important outcomes? Were benefits and costs that went beyond 1 year discounted (3% to 5%) and justification given for the discount rate? | 7 | x |  |
| 9. | Was the measurement of costs appropriate and the methodology for the estimation of quantities and unit costs clearly described? | 8 |  | x |
| 10. | Were the primary outcome measure(s) for the economic evaluation  clearly stated and did they include the major short-term, long-term, and negative outcomes? | 6 | x |  |
| 11. | Were the health outcomes measures/scales valid and reliable? If previously tested valid and reliable measures were not available, was justification given for the measures/scales used? | 7 |  | x |
| 12. | Were the economic model (including structure), study methods and analysis, and the components of the numerator and denominator displayed in a clear, transparent manner? | 8 |  | x |
| 13. | Were the choice of economic model, main assumptions, and limitations of the study stated and justified? | 7 | x |  |
| 14. | Did the author(s) explicitly discuss direction and magnitude of potential biases? | 6 | x |  |
| 15. | Were the conclusions/recommendations of the study justified and based on the study results? | 8 | x |  |
| 16. | Was there a statement disclosing the source of funding for the study? | 3 |  | x |
|  | **TOTAL POINTS** | **100** | 57 |  |

**Supplementary Table S3. Results of ECOBIAS assessment.**

Relevant to study? Yes (1) /No (0)/ Partly (0.5)/ Unclear (0)/NA

| **Author,Year** | **A1** | **A2** | **A3** | **A4** | **A5** | **A6** | **A7** | **A8** | **A9** | **A10** | **A11** | **B1** | **B2** | **B3** | **B4** | **B5** | **B6** | **B7** | **B8** | **B9** | **B10** | **B11** | **Score** |
| --- | --- | --- | --- | --- | --- | --- | --- | --- | --- | --- | --- | --- | --- | --- | --- | --- | --- | --- | --- | --- | --- | --- | --- |
| Fabbri, 2020 | 0 | 1 | 0.5 | 0 | 1 | 1 | 0.5 | 0 | 1 | 0 | NA | 1 | 1 | 1 | 1 | 1 | 1 | 0.5 | 1 | 1 | 1 | 0.5 | 71% |
| Simons, 2021 | 1 | 1 | 1 | 0 | 1 | 1 | 0.5 | 1 | 1 | 1 | NA | 1 | 1 | 1 | 1 | 1 | 1 | 0.5 | 1 | 1 | 1 | 0.5 | 88% |
| Simons, 2023 | 1 | 1 | 1 | 0 | 1 | 1 | 0.5 | 1 | 1 | 1 | NA | 1 | 1 | 1 | 1 | 1 | 1 | 0.5 | 1 | 1 | 1 | 0.5 | 88% |
| Mfumbilwa, 2024 | 1 | 1 | 1 | 0 | 1 | 1 | 0.5 | 1 | 1 | 1 | NA | 1 | 1 | 1 | 1 | 1 | 1 | 0.5 | 1 | 1 | 1 | 0.5 | 88% |
| Fu, 2025 | 1 | 1 | 0.5 | 0 | 1 | 1 | 0.5 | 1 | 1 | 1 | NA | 1 | 1 | 1 | 1 | 1 | 1 | 0.5 | 1 | 1 | 1 | 0.5 | 86% |
| Schofield, 2019 | 0 | 1 | 0.5 | 0 | 1 | 1 | 0.5 | 0.5 | 1 | 1 | NA | 1 | 1 | 1 | 1 | 1 | 1 | 0.5 | 1 | 1 | 1 | 0 | 76% |
| Stark, 2019 | 0 | 1 | 0.5 | 0 | 1 | 1 | 0.5 | 0.5 | 0 | 1 | NA | 1 | 1 | 1 | 1 | 1 | 1 | 0.5 | 1 | 1 | 0.5 | 0 | 69% |
| Crawford, 2021 | 1 | 1 | 1 | 0 | 1 | 1 | 0.5 | 1 | 1 | 0 | NA | 1 | 1 | 1 | 1 | 1 | 0.5 | 0.5 | 1 | 1 | 1 | 0 | 79% |
| Avram, 2022 | 1 | 1 | 0.5 | 0 | 1 | 1 | 0.5 | 1 | 1 | 1 | NA | 1 | 1 | 1 | 1 | 1 | 1 | 0.5 | 1 | 1 | 1 | 0 | 83% |
| Lavelle, 2022 | 1 | 1 | 0.5 | 0 | 1 | 1 | 0.5 | 1 | 1 | 1 | NA | 1 | 1 | 1 | 1 | 1 | 0.5 | 0.5 | 1 | 1 | 1 | 0 | 81% |
| Sanford Kobayashi, 2022 | 0 | 1 | 0.5 | 0 | 1 | 1 | 0.5 | 0 | 0 | 1 | NA | 0.5 | 1 | 0.5 | 0 | 0.5 | 0.5 | 0 | 1 | 1 | 0 | 0 | 48% |
| Rosenberg Friedman, 2025 | 0 | 1 | 0.5 | 0 | 0.5 | 1 | 0.5 | 1 | 1 | 0 | NA | 1 | 1 | 1 | 1 | 0.5 | 0.5 | 0 | 0.5 | 0.5 | 0 | 0 | 55% |

**Part A: Overall checklist for bias in economic evaluation**ECOBIAS Item A1: Narrow perspective bias

ECOBIAS Item A2: Inefficient comparator bias

ECOBIAS Item A3: Cost measurement omission bias

ECOBIAS Item A4: Intermittent data collection bias

ECOBIAS Item A5: Invalid valuation bias

ECOBIAS Item A6: Ordinal ICER bias

ECOBIAS Item A7: Double-counting bias

ECOBIAS Item A8: Inappropriate discounting bias

ECOBIAS Item A9: Limited sensitivity analysis bias (duplication, refer to B10)

ECOBIAS Item A10: Sponsor bias

ECOBIAS Item A11: Reporting and dissemination bias (not applicable, for trial-based analysis)
**Part B: Model-specific aspects of bias in economic evaluation**

ECOBIAS Item B1: Structural assumptions bias

ECOBIAS Item B2: No treatment comparator bias

ECOBIAS Item B3: Wrong model bias

ECOBIAS Item B4: Limited time horizon bias

ECOBIAS Item B5: Bias related to data identification

ECOBIAS Item B6: Bias related to baseline data

ECOBIAS Item B7: Bias related to treatment effects

ECOBIAS Item B8: Bias related to quality-of-life weights (utilities)

ECOBIAS Item B9: Non-transparent data incorporation bias

ECOBIAS Item B10: Limited scope bias

ECOBIAS Item B11: Bias related to internal consistency
